# 16S rRNA gene pyrosequencing of reference and clinical samples and investigation of the temperature stability of microbiome profiles

**DOI:** 10.1186/2049-2618-2-31

**Published:** 2014-09-16

**Authors:** Jun Hang, Valmik Desai, Nela Zavaljevski, Yu Yang, Xiaoxu Lin, Ravi Vijaya Satya, Luis J Martinez, Jason M Blaylock, Richard G Jarman, Stephen J Thomas, Robert A Kuschner

**Affiliations:** 1 Viral Diseases Branch, Walter Reed Army Institute of Research, Silver Spring, MD 20910, USA; 2 Department of Defense Biotechnology High Performance Computing Software Applications Institute, Telemedicine and Advanced Technology Research Center, US Army Medical Research and Materiel Command, Fort Detrick, MD 21702, USA; 3 Current address: Infectious Diseases Service, WRNMMC, Bethesda, MD 20889, USA; 4 Current address: Qiagen Sciences Inc, Frederick, MD 21703, USA

## Abstract

**Background:**

Sample storage conditions, extraction methods, PCR primers, and parameters are major factors that affect metagenomics analysis based on microbial 16S rRNA gene sequencing. Most published studies were limited to the comparison of only one or two types of these factors. Systematic multi-factor explorations are needed to evaluate the conditions that may impact validity of a microbiome analysis. This study was aimed to improve methodological options to facilitate the best technical approaches in the design of a microbiome study. Three readily available mock bacterial community materials and two commercial extraction techniques, Qiagen DNeasy and MO BIO PowerSoil DNA purification methods, were used to assess procedures for 16S ribosomal DNA amplification and pyrosequencing-based analysis. Primers were chosen for 16S rDNA quantitative PCR and amplification of region V3 to V1. Swabs spiked with mock bacterial community cells and clinical oropharyngeal swabs were incubated at respective temperatures of -80°C, -20°C, 4°C, and 37°C for 4 weeks, then extracted with the two methods, and subjected to pyrosequencing and taxonomic and statistical analyses to investigate microbiome profile stability.

**Results:**

The bacterial compositions for the mock community DNA samples determined in this study were consistent with the projected levels and agreed with the literature. The quantitation accuracy of abundances for several genera was improved with changes made to the standard Human Microbiome Project (HMP) procedure. The data for the samples purified with DNeasy and PowerSoil methods were statistically distinct; however, both results were reproducible and in good agreement with each other. The temperature effect on storage stability was investigated by using mock community cells and showed that the microbial community profiles were altered with the increase in incubation temperature. However, this phenomenon was not detected when clinical oropharyngeal swabs were used in the experiment.

**Conclusions:**

Mock community materials originated from the HMP study are valuable controls in developing 16S metagenomics analysis procedures. Long-term exposure to a high temperature may introduce variation into analysis for oropharyngeal swabs, suggestive of storage at 4°C or lower. The observed variations due to sample storage temperature are in a similar range as the intrapersonal variability among different clinical oropharyngeal swab samples.

## Background

Bacteria are the most abundant and genetically diverse organisms, which ubiquitously inhabit the environment including many extremely adverse environments. Billions of bacteria exist in various locations on the human body as either commensal microbial flora, transient dwellers, or even opportunistic pathogens capable of causing acute or chronic infections [1-10]. The importance of healthy microbiota for human well-being and the association between human microbiome and diseases have been shown in various studies, including colon cancer [11-13], obesity [14,15], and type II diabetes [16,17].

The use of advanced high-throughput techniques, such as microarrays and next-generation sequencing (NGS), has led to an explosive accumulation of research data and has vastly improved our understanding of the microbial world [7,18,19]. The Human Microbiome Project (HMP) funded by the National Institutes of Health has produced critical baseline information on healthy human microbiota and has also added a variety of metagenomics laboratory protocols and bioinformatics tools (http://www.hmpdacc.org) [5,20]. For metagenomics studies based on 16S ribosomal RNA gene (rDNA) sequencing, reliable procedures for sample collection, nucleic acid extraction, PCR amplification, amplicon sequencing, and data analysis are critical for the accuracy and resolution of quantitative and comparative study on microbial communities [18,21,22]. There have been reports on characterization of reference metagenomics materials and comparison of specimen storage conditions and optimization of methods [23-27]. However, most published studies were limited to the comparison of variable conditions of only one or two types of these factors. Systematic explorations of multiple factors are needed to evaluate the conditions that may impact validity of a microbiome analysis. In this study, we used the mock bacterial community genomic DNA samples and the mock bacterial community cells, both of which originated from the HMP [5,27,28], to test laboratory and data analysis procedures that will be applied to a population study of human respiratory microbiomes. Moreover, this pilot study was developed specifically to evaluate technical options which have not been investigated. Swabs spiked with the mock community bacterial cells and the clinical throat swabs from healthy human subjects were stored at four different temperatures for 4 weeks and sequenced to assess the durability of the microbiome profile over time and at various storage temperatures.

## Methods

### Microbial mock communities

**Figure 1:**
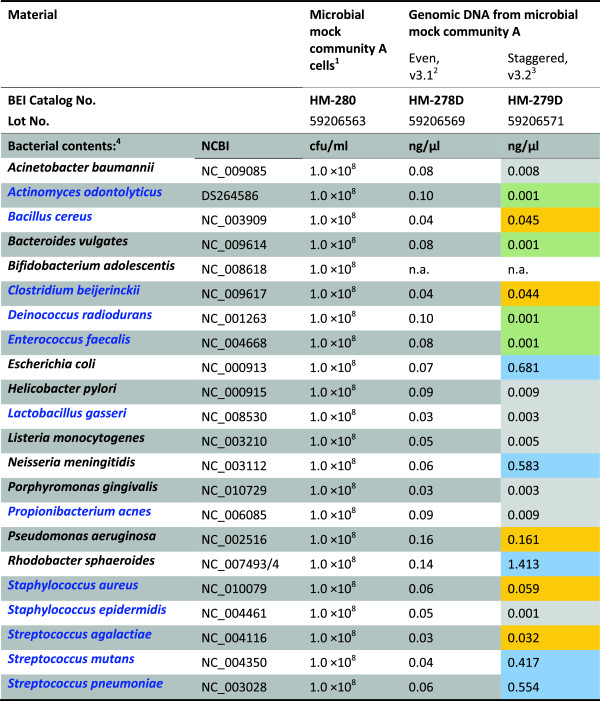
**Microbial mock community reference materials from BEI Resources used in the study to evaluate laboratory and data analysis procedures.** The sample information was referred from product information sheets or certificates of analysis for the materials. (*1*) A cell mixture of 22 different bacterial species was made to contain 1 × 10^8^ colony-forming units/ml (cfu/ml) of each species. (*2*) A mixture of genomic DNA from 21 different bacterial species. Individual DNA extracts were quantified by using Qubit Fluorometer and mixed based on the genome size and the copy number of 16S ribosomal RNA genes in each genome to have equal-molar 16S rDNA copies for each species. (*3*) A mixture of genomic DNA from 21 different bacterial species containing unequal-molar 16S rRNA genes for each species. Individual DNA concentrations were determined by Qubit Fluorometer. Species with relative abundance (16S rDNA copy) of approximate 20%, 2%, 0.2%, and 0.02% were indicated by shading in *blue*, *orange*, *gray*, and *green*, respectively. (*4*) Gram-negative and Gram-positive bacteria are shown in *black* and *blue fonts*, respectively.

Three microbial mock community materials (Figure 1) were obtained from Biodefense and Emerging Infectious Research (BEI) Resources of the American Type Culture Collection (ATCC) (Manassas, VA, USA), including microbial mock community A cells (BEI catalog number HM-280), a cell mixture of 22 different bacterial strains with equal colony-forming unit (cfu) for each organism; a mixture of genomic DNA from 21 bacterial strains (BEI catalog number HM-278D), i.e., all species but *Bifidobacterium adolescentis* of the microbial mock community A, containing equal molar (even) of rRNA operon counts for each organism; and a mixture of genomic DNA from 21 bacterial strains containing rRNA operon counts different by up to 1,000-fold (staggered) (BEI catalog number HM-279D).

HM-280 was diluted to a final volume of 5 ml by adding 4 ml of phosphate-buffered saline (PBS) to 1 ml of HM-280. The final bacterial cell concentration was approximately 4.4 × 10^5^ cfu/μl. Forty microliters of this cell suspension was spiked on each Copan flocked swab, FLOQSwab tube 560C (COPAN Diagnostics Inc., Murrieta, CA, USA). Each swab was returned to the tube, recapped, and stored dry without using any storage solution. Forty-eight spiked swabs were made to investigate storage temperatures, microbiome stability upon storage, and extraction methods (Additional file 1: Figure S1). Triplicate swabs were made for each condition. The swabs were randomly divided into four groups, then incubated under four different temperatures (37°C, 4°C, -20°C, or -80°C), respectively, for 4 weeks.

### Collection and storage of clinical swabs

The clinical specimens used in this study were obtained under the terms of a human use protocol (WRAIR#1913), approved by the Walter Reed Army Institute of Research Institutional Review Board in compliance with all US federal regulations governing the protection of human subjects. Written, informed consent was obtained from the participants. Healthy young volunteers were recruited for the study. Four different regions of the oropharynx, upper right, upper left, lower right, and lower left, were swabbed using Copan flocked swabs. Each swab from each of the eight individuals was recapped and stored under four different temperatures (37°C, 4°C, -20°C, or -80°C), respectively, for 4 weeks.

### DNA extraction from the swabs

After incubation, the swabs were extracted using one of the two DNA extraction methods, PowerSoil DNA Isolation Kit (MO BIO Laboratories, Inc., Carlsbad, CA, USA) and DNeasy Blood & Tissue Kit (Qiagen, Germantown, MD, USA). In brief, for extraction using the PowerSoil DNA Isolation Kit, the swab tip was cut into a PowerBead tube containing 0.7-mm garnet beads using a clean blade and extracted according to the instruction manual. Bead beating for 3 min on Mini-Beadbeater-16 (BioSpec, Bartlesville, OK, USA) was used to facilitate cell lysis. Alternatively, DNeasy Blood & Tissue Kit was used to purify DNA from the swab. Prior to DNeasy extraction, the swab tip was cut into a clean microfuge tube and subjected to enzymatic lysis of bacterial cells as follows: 450 μl of pre-chilled enzymatic lysis buffer containing 1 mg/ml lysozyme (L6876, Sigma, St. Louis, MO, USA); 0.1 mg/ml lysostaphin (L9043, Sigma); 20 mM Tris-HCl, pH 8.0; 2 mM EDTA; and 1 mM DTT were added and mixed by shaking at 1,400 rpm for 1 min. The tube was incubated at 37°C for 60 min. Then 25 μl of Proteinase K solution (10 mg/ml, Qiagen) and 500 μl of Buffer AL of DNeasy kit were added and mixed again at 1,400 rpm for 1 min, followed by incubation at 56°C for 2 h. After vigorous mixing via a vortexer or the beadbeater, the solution was collected and centrifuged at 13,000×*g* for 1 min. The supernatant was then processed by following the protocol in the DNeasy handbook to purify the total DNA.

### Quantitative PCR for 16S rDNA, amplification, and pyrosequencing of 16S rDNA region V3 to V1

**Figure 2:**
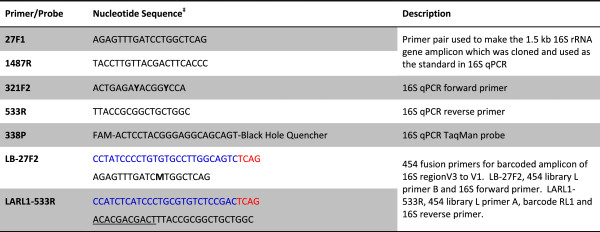
**Oligonucleotides used in the study.** The nucleotide positions for the primers and probe were numbered corresponding to the 1,542-bp 16S rRNA gene (*rrn*A) for *Escherichia coli* str. K-12 substr. MG1655 (NC_000913, nucleotide 4035531 to 4037072). ^‡^Degenerate nucleotides are shown in *bold*, **Y** for C or T, **M** for A or C. Sequences in *blue*, 454 sequencing system primers A or B. Sequences in *red*, 454 sequencing key for amplicon sequencing. The *underlined* sequence is the barcode, for which 454 Rapid Library Multiplex Identifiers (RLMIDs) was used. RL1 is shown as an example.

The purified total DNA samples were subjected to a real-time PCR assay to assess the nucleic acid extraction yield. Primers and TaqMan probe (Figure 2) were designed to target conserved sequences around the variable region 3 (V3) of bacterial 16S rDNA. Genomic DNA of *Staphylococcus aureus* strain TCH70 (HM-139D, BEI) and primers 27F1 and 1487R (Figure 2) were used to generate the 1.5-kb 16S rDNA amplicon which was subsequently cloned into vector pCRII-TOPO (Invitrogen, Carlsbad, CA, USA). The plasmid was used as a standard for the quantitative PCR (qPCR) using Taqman Universal Master Mix II (Invitrogen) to determine 16S rDNA copy number in DNA samples. PCR fusion primers LB-27F2 and LARL-533R for amplification and barcoding of the 16S rDNA region from its variable region V3 to V1 were designed following the HMP protocol with modification (Figure 2). The choice of the most conserved 16S rDNA sequences for qPCR or PCR primers and the use of degenerate bases were made by using the command line version of Primer3 software [29] installed on a local Linux server and published literature and resources [29-33]. The specificity/universality of candidate primers to 16S rDNA sequences was tested using the Ribosomal Database Project (RDP) probematch utility (http://rdp.cme.msu.edu/probematch/search.jsp) [34]. Optimization of primer specificity/universality was conducted by using one or two degenerated bases. The primers were tested using the greengenes probe locator tool (http://greengenes.lbl.gov/cgi-bin/nph-probe_locator.cgi) to determine where and which degenerated bases to be used. The modified primers were subsequently retested using the RDP probematch utility to verify the improvement. Furthermore, free energy calculations were performed using the Quickfold program from the DINAMelt Web server (http://mfold.rna.albany.edu/?q=DINAMelt/Quickfold) to eliminate primers that form stable 2D structures. For the final choice of primers for qPCR or PCR for producing sequencing amplicons, forward primers and reverse primers were paired manually based on the desired amplicon length and keeping the melting temperature (*T*_m_) difference between the two primers within 5°C.

The PCR procedure for generation of 16S V3–V1 amplicons was the same as in the HMP protocol [35], except that the PCR cycle number was set based on the Ct value from the 16S qPCR assay. Rather than using a fixed 30-cycle PCR for every sample, a cycle number of 20, 25, or 30 was chosen for each sample individually, based on the Ct value of a sample. The amplicons were purified using Qiagen's QIAquick 96 PCR purification kit, quantified using Quant-iT PicoGreen dsDNA assay (Invitrogen), and then pooled together at equal molar ratio. The pool of the amplicons was subjected to agarose gel size selection by electrophoresis using SizeSelect 2% E-Gel (Invitrogen), recovering the fraction in the size range of 500–1,000 bp using the disposable x-tracta gel extraction tool (Sigma). The amplicons were recovered by using QIAquick gel extraction kit, followed by DNA quantitation and quality examination using 2100 Bioanalyzer and the High Sensitivity DNA Assay kit (Agilent Technologies, Santa Clara, CA, USA). The final amplicon preparation products were used in emulsion PCR via Roche GS Lib-L LV kit (454 Life Sciences Corporation, Branford, CT, USA) with the use of molecules-per-bead ratio of 0.83 and 57.5 μl of amplification primer mix in the 3,915 μl reaction mix. The emulsion PCR, library bead purification, and sequencing on Roche 454 GS FLX+ system were performed by following the manufacturer recommended protocols.

### Pyrosequencing data processing and taxonomic classification

**Figure 3:**
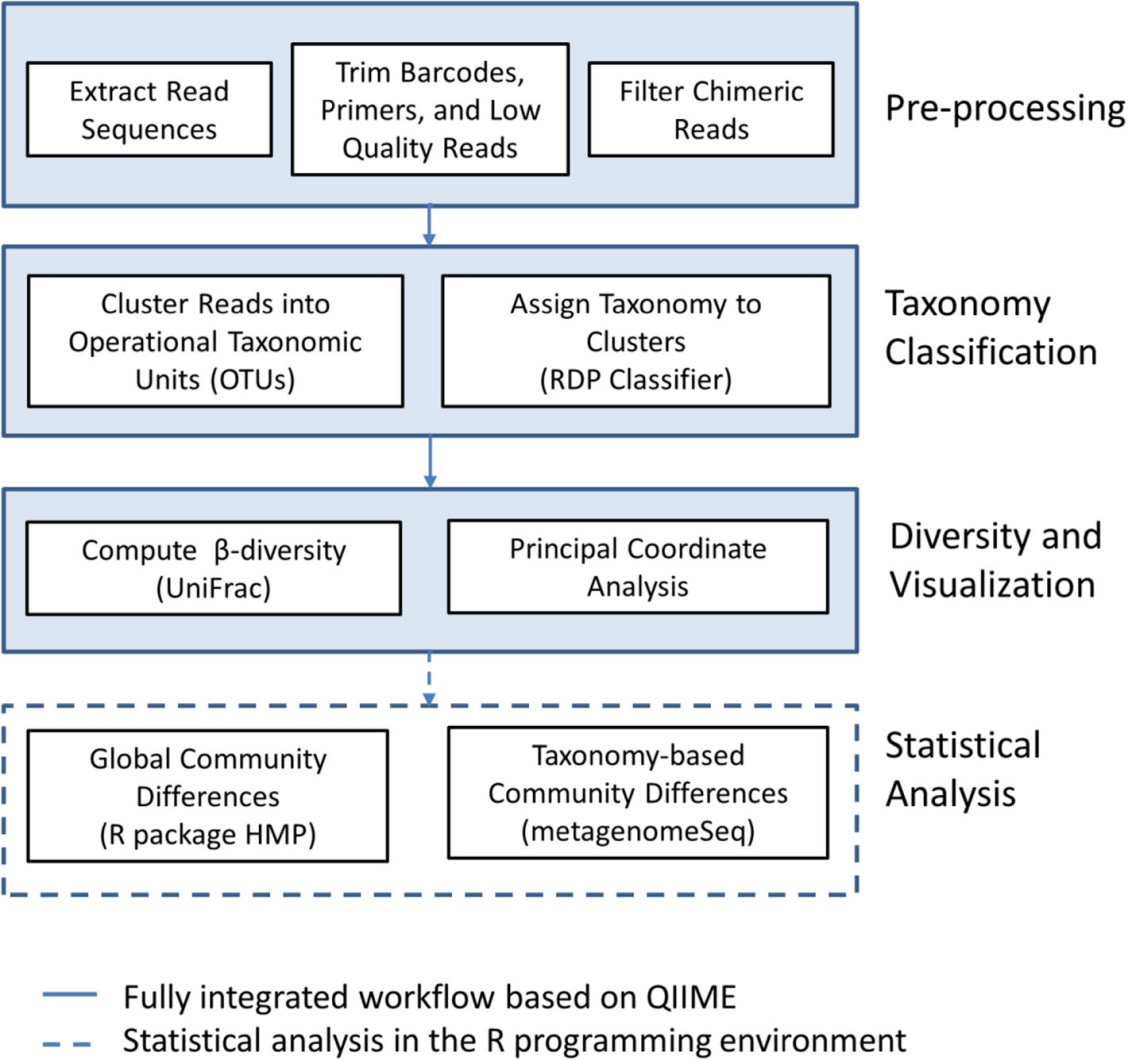
**Workflow for data analysis of the respiratory microbiome.** QIIME-based analysis is performed in three steps: (1) pre-processing, (2) taxonomy classification, and (3) computation of diversity and visualization. Further statistical analysis of the data is performed in R programming environment.

A data analysis workflow based on the Quantitative Insights Into Microbial Ecology (QIIME) pipeline was implemented (Figure 3) [36]. Pyrosequencing data sff file was demultiplexed with the barcode mismatch tolerance of one base for the 11-base Molecular Identifier (MID) tags. Raw reads were subjected to a quality filtering procedure in the following consecutive steps: terminal trimming to remove N from the 3′-end of the raw reads, removal of reads that are smaller than 200 bases or larger than 1,000 bases, removal of reads that have homopolymer eight bases or longer, removal of reads that contain more than one error in the 16S primer 539R sequence, read trimming to remove primer and linker sequences, sliding window trimming with a window width of 50 bases to remove the terminal sequence within the window with an average quality score below 25. Chimera filtering was performed afterwards using the UCHIME algorithm by either reference-based or *de novo* method [37]. Reads that were classified as chimeric by both methods were removed. Finally, singleton reads were excluded from further analysis. For bacterial taxonomic classification, the quality processed reads were subjected to analysis using the QIIME pipeline run by Python programs. The workflow included open-reference clustering of sequences into operational taxonomic units (OTUs) using the UCLUST tool. The sequence identity level was set at 97%, which corresponds to a commonly used bioinformatics definition of the bacterial species based on the 16S rRNA gene. The read clusters were further assigned to taxonomies using the RDP classifier with the confidence level of 80% [34]. The microbial profiles obtained after this step contained various hierarchical levels of taxonomy classification, and their positions in the taxonomy were used to assess diversity for each community. In the statistical analyses, the reads assigned to taxonomy levels below the genus level were mapped to the corresponding genus level for further evaluation of statistical significance at the genus level.

### Microbiome diversity estimation and statistical analysis

The genus-level microbiome profiles from QIIME/RDP analysis were used to evaluate the microbial community diversity within a sample (α-diversity) and the diversity between samples (β-diversity). Tools for variability analysis in QIIME, including the comparison of abundance of microbial taxa present in the samples, weighted UniFrac measure, and the multidimensional principal coordinate analysis (PCoA), were used [38]. Two recently proposed methods were collaboratively used for multinomial statistical analysis of the microbiome data. The statistical analysis consisted of three steps: (1) for each microbiome community, use the R statistical software package for HMP (HMP-R) by La Rosa et al. [39,40] to test the underlying probabilistic model based on the Dirichlet multinomial (DM) distribution and to determine the DM parameters, proportions, and dispersion [39]; (2) use the HMP-R to perform hypothesis testing of overall significant differences between communities; and (3) use the R software package metagenomeSeq to determine OTUs that are statistically different in the two communities [41,42].

## Results and discussion

### Primers, PCR amplification, and 454 pyrosequencing

The V1–V3 region of the 16S rRNA gene was used in most 16S rDNA sequencing-based metagenomics studies and was also chosen in this study. Instead of using 16S primers 27F1 (AGAGTTTGATCCTGGCTCAG) and 534R (ATTACCGCGGCTGCTGG) in the HMP procedure [35], we utilized the enormous 16S rDNA sequence data rapidly accumulated in recent years to search for primers which provide the best match to most identified bacteria. The resulted primers 27F2 and 533R (Figure 2), though very similar with 27F1/534R and reported in other studies [31,43], had two differences from the HMP V1/V3 primer pair: the use of degenerated base M in 27F2 instead of base C and the sequence shift by one base toward 5′-end from 534R to 533R. These changes in primer design led to increased percentage of 16S sequences matching to the primers with none or one-base mismatch (Additional file 2: Table S1). As a result, we saw improved representation of bacteria, such as *Acinetobacter baumannii*, *Escherichia coli*, and *Pseudomonas aeruginosa* in the mock bacterial community A (data shown below).

**Figure 4:**
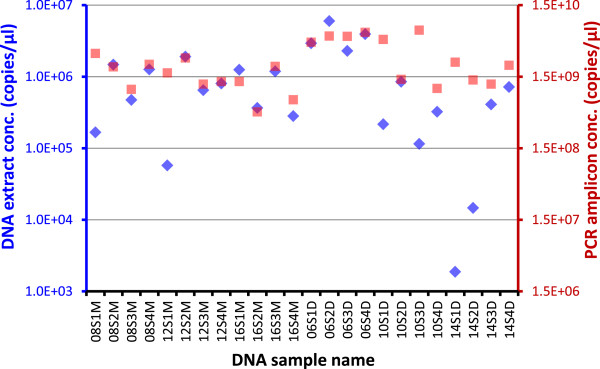
**Normalization of PCR amplification by choosing PCR cycle number close to the Ct value.** Throat swabs from healthy volunteers were extracted with Qiagen DNeasy (D) or MO BIO PowerSoil (M). DNA extracts were subjected to 16S gene TaqMan qPCR and subsequent PCR using a cycle number of 20, 25, or 30 based on individual sample's qPCR Ct value. PCR amplicons were quantified by PicoGreen dsDNA assay. DNA concentrations for DNA extracts (*blue markers and axis*) and PCR amplicons (*red markers and axis*) are shown in 16S gene copies/μl in the same scale. DNA sample name, 08S1M as an example, depicts the subject number (08), swab number (S1), and extraction method (M).

PCR amplification variability and biases are potential causes for data errors [21,44]. To verify the data reproducibility for the Roche GS FLX+ system used in the study, variations among technical replicates were evaluated. The replications tested included three PCR reactions prepared and amplified independently, duplicated emulsion PCR reactions and bead preparations, loading sequencing beads of the same preparation on different regions on a 454 picotiter plate, and sequencing the mock community on different 454 runs. In the HMP procedure, DNA extracts were quantified by fluorescent assay and 2 μl of either 1:1 diluted or undiluted DNA of each sample was used in the PCR amplification with the cycle number of 30 used for all samples. In this study, we did qPCR assay to determine 16S rDNA copy numbers in DNA extracts and found that the concentrations (copies/μl of 16S rDNA) for human throat swabs from this study (*n* = 32) were highly variable, with the concentrations varying from below 10^3^ to over 10^6^ copies/μl (Figure 4 blue markers) and Ct values from approximately 15 to >25 from swab to swab. Therefore, instead of using 30 cycles for all samples, we chose a cycle number of 20, 25, or 30 based on the qPCR Ct value for a particular sample. PCR amplification for most samples, regardless of their initial concentrations, was kept within the log phase, and consequently, the resulted amplicons had concentrations close to each other (Figure 4 red markers).

By setting the PCR cycle number based on the sample's 16S qPCR Ct value, i.e., no more than Ct +5, we were able to prevent the amplification from reaching PCR saturation. This might be a way to reduce one of the major sources for the PCR biases which are exacerbated when the genes are over-amplified. To ensure that amplicons by PCR with a lower PCR cycle number are suitable for pyrosequencing, we amplified the 1:10 diluted genomic DNA reference sample HM-278D, which had a Ct value of 18.9, for 25 cycles (Ct +6.1) and 20 cycles (Ct +1.1), respectively, in duplicate, applied to emulsion PCR (emPCR) and the beads were loaded in three regions of a four-region picotiter plate, named as 25.1, 25.2, 25.3 and 20.1, 20.2, 20.3, respectively. The microbial profiles for these replicates were obtained using the QIIME pipeline and evaluated at the genus level. They were compared to the ‘projected’ percentage rate (discussed below) for each component. Root-mean-square error for absolute differences for each genus was calculated, which was 60.24% (20.1), 56.20% (20.2), and 55.89% (20.3), respectively, for the 20-cycle amplicons (average 57.44% ± 2.43%) and 63.84% (25.1), 63.31% (25.2), and 64.90% (25.3), respectively, for the 25-cycle amplicons (average 64.02% ± 0.81%). There were no statistically significant differences (*P* value = 1) between any pair of these bacterial profiles from PCR of different cycle numbers, emPCR replicates, and picotiter plate regions. The correlation coefficient between average profiles for 20-cycle and 25-cycle amplicons was 0.995. Interestingly, none of these profiles statistically resembled the projected composition (Additional file 3: Table S2), with the *P* values corrected for multiple comparisons between 0.006 and 0.024. In addition, the concentration differences for the PCR products as determined by PicoGreen dsDNA assay were just about 10-fold (Figure 4). This close proximity of amplicon concentrations not only facilitated equal molar ratio pooling of PCR products but also appeared to result in similar number of sequence reads produced for each sample in the pool.

### Pyrosequencing data processing and reads classification for mock community DNA

**Figure 5:**
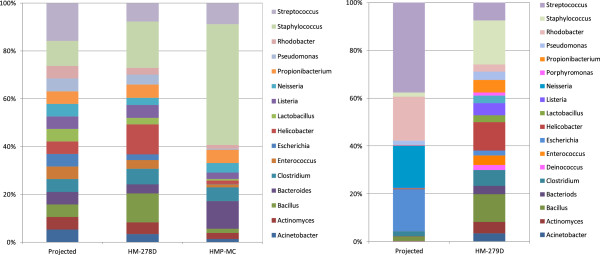
**Relative bacterial abundance determined by OTU from 454 pyrosequencing analysis.** HM-278D and HM-279D were amplified by PCR for 20 cycles, respectively. Data for mock community DNA equal-molar mix used in HMP studies (HMP-MC) were from the HMP Data Analysis and Coordination Center (DACC) and NCBI. All data were analyzed using the QIIME-based pipeline, with classification of operational taxonomic units (OTUs) to bacterial genus level. *Left panel*, mock community DNA equal-molar mix. *Right panel*, mock community DNA staggered-molar mix. *Projected*, the relative bacterial abundance calculated based on DNA quantities used in making the HM-278D and HM-279D.

An equal-molar mixture of genomic DNA for a mock community (HMP-MC) consisted of 20 bacterial species and one archaebacterium, was extensively evaluated, and was used as positive controls in the HMP studies [28]. The even mock community sample HM-278D used in this study is derived from the HMP study and equivalent to HMP-MC. These two were produced in the same way and contain comparable bacterial composition. HM-278D contained all the bacterial species in the HMP mock and an additional species of *Porphyromonas gingivalis*, but has no archaebacterium. The amount of *Deinococcus* added was increased more than 10 times in HMP-MC because of its exceedingly low Ct value in the qPCR assay (Additional file 3: Table S2) [5]. The data for HMP-MC from the HMP database and the data for HM-278D in this study were both processed and analyzed by using the QIIME pipeline and the parameters we chose for this study. The bacterial profiling results for HMP-MC obtained by the QIIME pipeline (HMP-MC average relative abundance in Additional file 3: Table S2) were similar to the published results obtained by the mothur pipeline [45] (V13 in Figure four of Schloss et al. [28]). The QIIME analysis results for HM-278D and the HMP-MC data were compared and are shown in Figure 5 and Additional file 3: Table S2. Sequence reads for genera *Porphyromonas* and *Deinococcus* were not included in this comparison. For 13 out of 16 genera in the analysis, the relative bacterial abundances as compared to the projected levels were consistent between HM-278D and HMP-MC, i.e., for each of the 13 genera, the relative abundance which was shown above (or below) the projected level in HMP-MC results was also seen higher (or lower) than the projected level in HM-278D results. Large differences between HM-278D and HMP-MC results were seen in four genera—*Bacillus*, *Bacteroides*, *Helicobacter*, and *Staphylococcus*. We speculate that this inconsistency was because of the technical variations in the preparation of the bacterial genomic DNAs and the DNA mixtures. HMP-MC component genomic DNAs were quantified with 16S qPCR assay individually before pooling [5] whereas HM-278D component genomic DNAs were quantified with PicoGreen assay to estimate 16S rDNA copy number in each DNA extract for the pooling (Figure 1). It is not surprising that molar ratios for the components vary from lot to lot; therefore, mock community DNA from a same manufacture lot needs to be used in serial sequencing runs for a metagenomics study. As we expected, the use of the degenerate base M instead of C in 16S primer 27F2 (Figure 2) significantly elevated the relative abundances for *A. baumannii*, *E. coli*, and *P. aeruginosa* which were all barely detectable when primer 27F1 was used [28]. Moreover, the overall difference as compared with the projected was smaller for HM-278D (root-mean-square error 56.50%) than HMP-MC (112.80%). Together, the results indicate that the procedure and analysis established in this study achieved comparable or improved performance compared to those in the HMP studies.

The pyrosequencing procedure was then used to evaluate the uneven mock community DNA (HM-279D), which contains 21 bacterial species with 16S gene copy numbers staggered from 1,000 copies/μl (*n* = 4), 10,000 copies/μl (*n* = 7), 100,000 copies/μl (*n* = 4) to 1,000,000 copies/μl (*n* = 6), and correspondent varied relative abundance of 0.02%, 0.2%, 2%, or 20% (Figure 1) [5]. Though described as a HMP resource and distributed through BEI for research use, the sequencing data and the analysis for this uneven mock community were not available in either database or publication. It may be a suitable reference material of a complex community with highly variable component abundances if a result consistent with the formula can be reliably produced. To investigate the relative bacterial abundances, the level of results variation and reproducibility, and the limit of detection, 1:10 diluted HM-279D was amplified by PCR for 20 cycles in triplication and sequenced. The results were reproducible among PCR and sequencing replicates (with a correlation coefficient greater than 0.99) and in good agreement with the bacterial composition of HM-279D (with a correlation coefficient larger than 0.9). However, individual genera were detected with a variable degree of reproducibility, depending on the abundance (Figure 5, Additional file 3: Table S2). The most abundant (about 20% or higher) and the abundant (about 2%) genera were readily detected (7/8), except for *Pseudomonas*, which had a projected abundance of 1.82% but was seen only 0.12% in the results (Additional file 3: Table S2). The low abundant (0.2%) genera could be detected, yet there were significant differences between the results and the projected abundance (Figure 5, Additional file 3: Table S2). It is not surprising that most of the exceedingly low (0.02%, i.e., 2 in 10,000 reads) contents were undetectable (3/4) when 5,000–10,000 reads were obtained for the analysis. Similar results were seen in the repeated experiments. The poor reproducibility of the results in the low abundance range and the large difference in results with the projected composition (Additional file 3: Table S2) are consistent with the assessments of human microbiota [46]; deteriorated accuracy and uncertainty in quantitation of low abundant microbes has been observed in most studies using clinical specimens. Together, these data suggest that it may not be necessarily informative to include the uneven mock community DNA HM-279D as a quality control to verify sensitivity and reliability for detecting low-abundance bacteria in a complex community.

### Comparison of DNA extraction methods and storage temperatures

**Figure 6:**
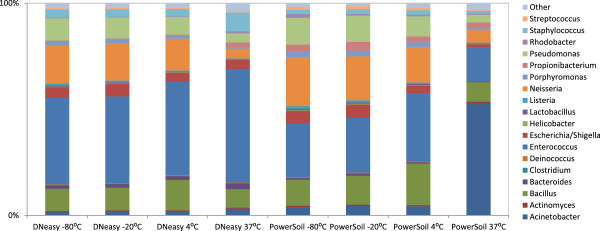
**Storage temperature comparisons for Qiagen DNeasy and MO BIO PowerSoil DNA extraction methods for the mock bacterial community HM-280.** Identical samples from the mock community were stored at four different temperatures and extracted after 4 weeks using the two methods, Qiagen DNeasy and MO BIO PowerSoil. Microbial profiles at the genus level were estimated using QIIME. Overall, samples were well preserved at lower temperatures for both methods, whereas significant differences were observed at 37°C.

**Figure 7:**
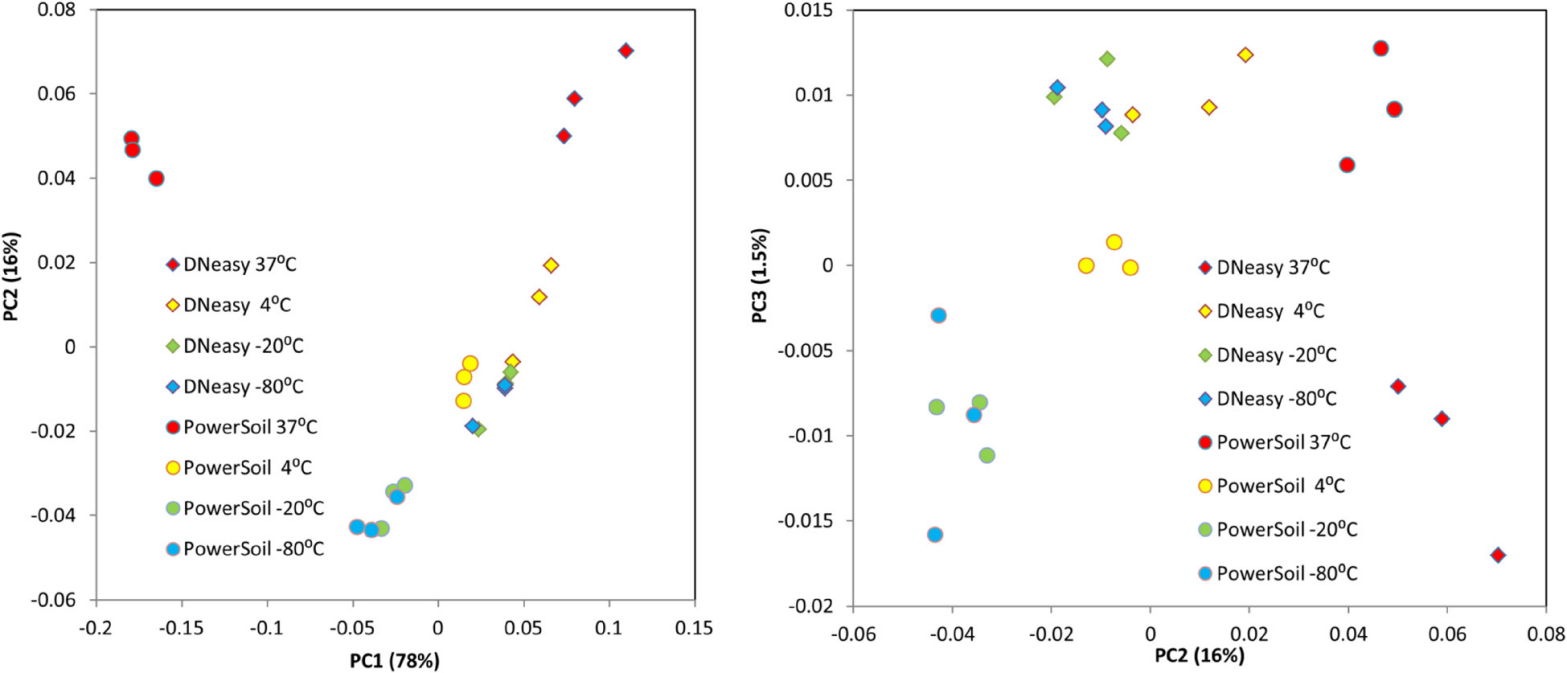
**Diversity of microbiome profiles with storage temperatures and extraction methods for the mock bacterial community HM-280.** The principal coordinate analysis is based on the weighted UniFrac distances between the microbiome profiles. Equal aliquots of the mock bacterial community were stored for 4 weeks at four different temperatures, extracted by two methods, and amplified in three PCR replicates. Each coordinate axis explains the specified percent of the total community variability.

Qiagen DNeasy kit with additional enzymatic lysis and MO BIO PowerSoil kit with bead beating were both frequently used in metagenomics studies [24,47]. We compared these two methods for extraction of DNA from Copan flocked swabs spiked with mock bacterial community cells HM-280 (Additional file 1: Figure S1). DNA extracts were sequenced for evaluation of data variation and compositional differences between extraction methods (Figure 6, Additional file 3: Table S2). The mock bacterial community HM-280, though a mixture of bacterial cells with equal colony-forming units from each of the 22 species, was not quantified for true genome copy number for each component by the manufacturer. This study is the first report of quantitative assay and pyrosequencing of this mock community cells which might be a useful reference for assessment of an extraction method and a common control to be used across labs. The DNA yields by using the two methods, as determined with 16S qPCR, were comparable to each other and reproducible among respective replicates (Additional file 1: Figure S1). First, we analyzed sequencing data for swabs stored under -80°C to compare the two extraction methods. The results suggest high reproducibility for both methods. The correlation coefficients are greater than 0.98 for any pair of PCR or sequencing replicates for each method. The bacterial profiles for DNA extracts for the swabs stored at -80°C and extracted with these two methods at the storage temperature of showed similarity (11/18 genera with *P* > 0.05) and differences (7/18 genera with *P* < 0.05) in community composition as presented in Figure 6 and Additional file 3: Table S2 and Additional file 4: Table S3. In addition, on the β-diversity analysis plot in Figure 7, for both DNeasy -80°C and PowerSoil -80°C, the swabs, in triplicate, were clustered together indicating good technical reproducibility for both methods; however, the two clusters were clearly separated from each other, suggesting consistent discrimination between these two methods. Statistical analyses were performed to examine the detailed differences and to investigate the possible existence of consistent biases correlated to the extraction methods. The overall differences in the microbial communities stored at -80°C and extracted with the two methods were tested using the HMP-R software. The hypothesis of equal Dirichlet multinomial distributions for the two communities was rejected with *P* value = 0. Differences in each genus in the communities were tested using metagenomeSeq software. Statistically significant differences were indicated for 7 out of 18 genera, as presented in Additional file 4: Table S3. The relative abundances determined with extracts by using the two methods were in close range within 1- to 3-fold for most bacteria (16/18 genera), while for the abundances of *Rhodobacter* and *Propionibacterium* (2/18 genera), the differences were 4- and 17-fold, respectively (Additional file 3: Table S2).

To investigate whether there was a significant level of bacterial contamination in the materials which may contribute to the differences, we extracted, quantified, and sequenced bacterial 16S rDNA in swabs and extraction reagents to assess the presence of 16S genes. Nothing but the lysis beads used in MO BIO PowerSoil extraction was found to contain a few bacteria at low level. The concentrations for the non-spiked blank controls of PowerSoil extraction with or without the presence of a clean swab were varied by 600–1,600 copies/μl in the extracts, approximately 10–20 times higher than those for the controls of DNeasy methods. PCR results after 30-cycle amplification and sequencing were negative for the blank controls with DNeasy methods, while several bacteria including *Aeromonas*, *Gemmata*, *Haemophilus*, *Schwartzia*, *Propionibacterium*, *Sulfurospirillum*, and *Williamsia* were present in PowerSoil controls when the lysis beads were used. In the study by Willner et al*.*, five DNA extraction methods, including PowerSoil kit and NucleoSpin Tissue kit which is very similar with DNeasy, were tested for introduction of microbial contamination into DNA extracts. PowerSoil was one of the two methods that gave the highest level of contamination [47]. The level and contents of bacterial contamination coming from the manufacturer's production materials and facility may vary from lot to lot. The results suggest the necessity of including adequate controls in metagenomics studies, in particular when the bacterial contents in the subject are considerably low or rare microbes are of interest.

It has been shown that the microbial community profile can be well preserved in low freezing temperatures and may be subjected to various changes over time under suboptimal conditions [48-50]. On the contrary, some results show that microbiome profiles were fairly well preserved upon 2 weeks storage under 20°C [23] or for at least 24 h at room temperature [51]. In this study, the mock community bacterial cells HM-280 from BEI were used to investigate the impact of storage temperature on microbial profile stability. We tested swabs spiked with an equal amount and composition of bacterial cells to compare four storage temperatures (37°C, 4°C, -20°C, and -80°C). Both DNeasy and PowerSoil extraction methods were used in the investigation. The 16S quantitative PCR results clearly indicated that 37°C incubation can cause substantial degradation of 16S rDNA, while 4°C or lower temperature storage may maintain 16S rDNA integrity for a long period of time (Additional file 1: Figure S1). Relative abundance analysis of bacterial genera (Figure 6, Additional file 4: Table S3) suggested the overall similarity of the microbiome profiles for different temperatures when the same extraction method was used. When compared with the results for swabs stored in -80°C, increased divergence in the microbial profiles was seen with increased storage temperature. In particular, 37°C incubation led to much higher divergence as compared with freezing or refrigeration conditions.

We tested preservation of the community structures using the statistical package HMP-R to analyze the probability distributions of these communities. We tested the null hypothesis of the multinomial distribution (zero dispersion) against the DM distribution (non-zero dispersion) and rejected the null hypothesis with the *P* value = 0 in most cases. We then estimated the parameters of the DM distribution, the proportions of OTUs at the genus level, and the dispersion parameters for all samples. The estimated dispersion parameters can be used as measures of population variability. Larger dispersion indicates a lower detection probability for less abundant genera in the community. Estimated dispersion parameters for the mock bacterial community for different extraction methods and storage temperatures are presented in Table 1. Extracted DNA was PCR amplified three times, and sequenced in two different regions, giving a total of six replicates for statistical analysis. The dispersion parameters for samples of both extraction methods and all temperatures are of the order of 10^-3^. These are very small values compared to some reported dispersion values of the order of 0.01 for the respiratory microbiome [52] and to even larger dispersion parameters for the human microbiome, of the order of 0.1 [4]. Thus, it can be expected that the two extraction methods have similar probabilities to detect less abundant genera. Similar conclusions were made based on pairwise testing of the microbial communities, presented in Table 2. The overall community differences between the storage temperatures of -20°C and -80°C, regardless of the extraction methods used, were not significant. To assess a possibility to observe some differences in genera, we used metagenomeSeq to compute the pairwise differences on each genus for temperature -80°C versus the other temperatures, respectively (Additional file 4: Table S3). The quantitative test indicated that the microbial structure of the mock community was well preserved among samples stored in low temperatures, while long-term exposure to a high temperature, 37°C for 4 weeks in this study, resulted in substantial alterations in bacterial community structure.Additionally, variability of microbiome profiles for these communities was computed using UniFrac implemented in QIIME and is presented in Figure 7. The results confirmed that there was practically no difference in the first two principal components between the temperatures of -80°C and -20°C, and the communities at the temperature of 4°C are in close proximity to -80°C/-20°C communities, whereas the communities at the temperature of 37°C were clearly divergent from the communities at low temperatures.

**Table 1:** Dispersion parameters for the mock bacterial community

**Temperature (°C)**	**MO BIO PowerSoil**		**Qiagen DNeasy**	
	**Observed number of reads**	**Dispersion**	**Observed number of reads**	**Dispersion**
-80	5,056	1.49E - 03	6,215	2.53E - 03
-20	6,393	1.25E - 03	6,292	1.37E - 03
4	5,816	4.68E - 04	4,804	3.54E - 03
37	6,267	9.01E - 04	4,983	6.25E - 03

The Dirichlet multinomial (DM) distribution of OTU read counts is assumed. The DM parameters are OTU abundances and dispersion. DM dispersion represents a measure of community variability and is estimated using the R package HMP [39].

**Table 2:** Testing of the variation of the mock bacterial community with storage temperatures

	**MO BIO PowerSoil**			**Qiagen DNeasy**		
**Temperature (°C)**	**4**	**-20**	**-80**	**4**	**-20**	**-80**
37	0.00	0.00	0.00	0.00	0.00	0.00
4		0.00	0.00		3.23E - 11	7.18E - 9
-20			0.13			1.00

*P* values are used to test pairwise differences between communities. They correspond to the null hypothesis of equal parameters of the Dirichlet multinomial distribution models in the communities. *P* values are computed using the R package HMP [39]. *P* values less than the threshold 0.05 indicate significant differences between communities.

Taken together, by using mock community cells, we were able to compare the two widely used DNA extraction methods and four swab storage temperatures in detail. In agreement with a previous report [47], our results show that both Qiagen DNeasy and MO BIO PowerSoil extractions can produce statistically repeatable data for bacterial community analysis. However, despite the comparability between them, there are substantial discrepancies for some bacterial components in community when different extraction methods are used. The results strongly suggest that it is critical to maintain technical procedures consistent throughout the metagenomics study, and the technical differences need to be taken into consideration when comparing results from different studies. The use of mock bacterial community cells allowed accurate assessment of the association of storage temperature conditions with estimated bacterial community profiles on swabs.

### Microbiome analysis of clinical samples

**Figure 8:**
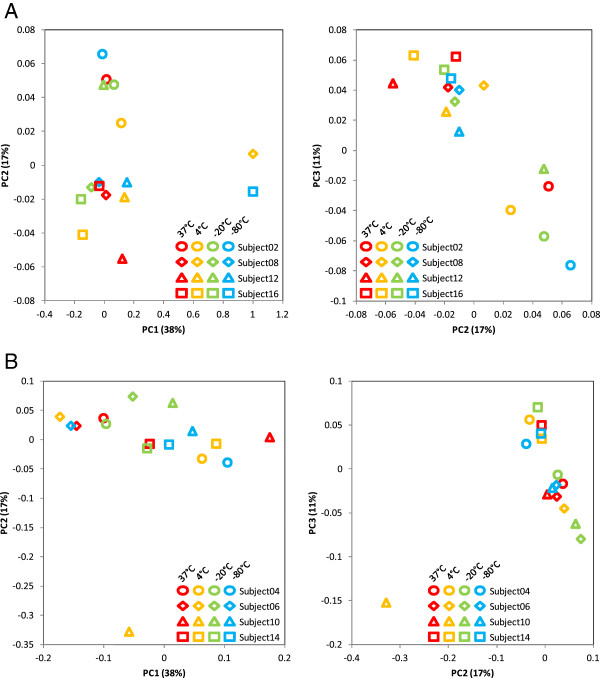
**Variability of oropharyngeal microbiome profiles with storage temperatures. (A)** Variability of oropharyngeal microbiome profiles with storage temperatures for the MO BIO PowerSoil extraction method. Four specimens were collected from each subject and stored for 4 weeks at different temperatures. Microbiome profiles at the genus level were estimated using QIIME. The figure presents the principal coordinate analysis based on the weighted UniFrac distances between these microbiome profiles. Each coordinate axis explains the specified percent of the total community variability. **(B)** Variability of oropharyngeal microbiome profiles with storage temperatures for the Qiagen DNeasy extraction method. Four specimens were collected from each subject and stored for four weeks at different temperatures. Microbiome profiles at the genus level were estimated using QIIME. The figure presents the principal coordinate analysis based on the weighted UniFrac distances between these microbiome profiles. Each coordinate axis explains the specified percent of the total community variability.

To explore whether the observations for the swabs spiked with the mock bacterial community cells were relevant for respiratory microbiome studies, we collected oropharyngeal samples from eight healthy volunteers. For each individual, four swabs from close but not overlapping spots in the throat were collected and each swab was stored at one of the four previously tested temperatures (37°C, 4°C, -20°C, and -80°C). After 4 weeks, DNA was extracted from the samples for four individuals using the DNeasy method and for the other four individuals using the PowerSoil method. The samples were PCR amplified in duplicate and analyzed using pyrosequencing and statistical data analyses. We used the weighted UniFrac measures of β-diversity to analyze the microbiome profiles, as presented in Figure 8. There was no apparent clustering of samples on either human subjects or storage temperatures. A pairwise comparison of storage temperatures for each subject was performed, and the results indicated significant changes (*P* value = 0) between throat swabs of any two temperatures which were from the same human subject. All but one swab samples contained the most common genera which were found abundant in oropharynges of healthy individuals [53]. *Streptococcus* was the most abundant one and varied from 28.5% to 76.5% in different individuals. The others included *Veillonella*, *Actinomyces*, and *Prevotella*. The only exception was a clear outlier: a swab sample from subject 10, stored at 4°C and extracted using the DNeasy method. The pairwise comparison of this sample with the samples from the same individual stored at other temperatures showed that this sample had high abundance of *Pseudomonas* (45.1%), *Serratia* (family *Enterobacteriaceae*) (13.2%), and *Yersinia* (family *Enterobacteriaceae*) (5.0%). Since all clinical samples were collected, processed, and analyzed in parallel by using similar materials and laboratory and bioinformatics procedures, and the human subject use protocol does not allow us to identify the subject for detailed clinical records or a repeated test, there was not enough evidence for us to determine the cause of this surprising difference—whether they were really present in individual oral specimens or from an accidental sample contamination. While a high concentration of *Pseudomonas* is fairly unusual in a healthy subject, its presence as a colonizer of the respiratory tract is well known. In addition, *Pseudomonas* has been described in prior microbiome studies as well. In a comparative study of nostril and oropharynx microbiota from seven healthy adults, *Proteobacteria*, the phylum which genus *Pseudomonas* belongs to, were found exceedingly abundant in three individuals' oropharyngeal samples while low in other four oropharyngeal samples and all seven nostrils [54].

The temperature effect observed for mock community cells was not observed in clinical oropharyngeal samples; in particular, the samples at 37°C did not appear to be distinguishable from those at low temperatures in the UniFrac β-diversity analysis (Figure 8). The seemingly discrepant observation is intriguing and worth a discussion. In contrast to the common variables including swab storage conditions, extraction methods, and PCR settings, the extent of variations among samples from the same individual (swab-to-swab variation) and variations between individuals (subject-to-subject variation) are more specifically related with microbial habitats and may be quite different from study to study. We noticed that most studies on storage conditions used liquid or solid specimens, such as stools, soils, or sputum, which can be thoroughly mixed and divided into homogenous subsamples to minimize sample composition difference. In this study, four throat swabs were collected from each individual and processed independently; therefore, the swab-to-swab intrapersonal variation was expected to be larger than the variations between mock community swabs or homogeneous subsamples. Despite all the differences in data analysis and interpretation, the results from various studies consistently indicate that variations between sample replicates from the same microbial habitat are substantially larger than those between technical replicates, but significantly lower than inter-individual differences and the differences between distinctive microbial habitats, e.g., body sites. Interestingly, in these studies, which used only a few samples, inter-subject differences were so large that the relatively smaller variations that resulted from storage condition changes did not lead to considerable blurring of separation of the subjects in the β-diversity analyses [23]. Oropharyngeal swabs in this study were collected from healthy individuals who work in the same environment regularly and who may subsequently possess a similar respiratory microbiome profile. We did not see consistently shorter UniFrac distances between -80°C and -20°C samples compared to the distances between -80°C and other temperatures. The distances between -80°C and -20°C samples, which can be taken as intrapersonal differences in oropharyngeal microbiome because it was shown in the mock community cells experiment that these two temperatures do not introduce marked variation, were greatly variable from person to person and, more importantly, were not always less than the interpersonal differences (Figure 8). Analyses suggested that healthy human oral habitats including the throat have relatively more even bacterial communities as compared with other body sites [4], although these may be less stable over time [9]. We assume that the participants from a close community might have a similar oropharyngeal microbiome. In these cases, the variations due to storage temperature conditions might be within the range of intrapersonal variability. However, our observations might be confounded by additional variability introduced due to the difficulties in dividing and homogenizing the throat sample from the same swab. We are cautious about whether uncontrolled sample storage temperature conditions will complicate comparative metagenomics analysis of the respiratory microbiome of a close community. Especially for long-term temporal microbiome dynamics or the investigation comparing samples collected from sites where the ambient temperatures are greatly variable, the temperature fluctuation may play a significant role and a pilot study on microbiome temperature stability might be warranted.

## Conclusions

In this study, we performed systematic and comprehensive characterization of three HMP reference materials from BEI Resources. Moreover, the mock bacterial community and oropharyngeal swabs from healthy individuals were used to investigate the temperature stability of the microbial community structure. The standard HMP procedure was optimized to include sequence modification of 16S rDNA primers for improved amplification of the V1–V3 region to increase coverage of bacterial 16S sequences in database, and the use of PCR cycle number related to 16S gene copy number in DNA extract to avoid over-amplification and to obtain PCR products with concentrations close to each other from samples with highly different concentrations. Pyrosequencing data of samples extracted by using the two popular methods, Qiagen DNeasy with enzymatic bacterial lysis and MO BIO PowerSoil with bead beating, are statistically different, however lead to consistent conclusions. The results for the even mock community DNA are consistent with a previous HMP report, with improvements which may be attributed to the technical changes. The temperature stability study using the assembled bacterial community suggests that the microbial community structure is stable at low temperature and may change significantly when incubated in high temperature. For studies on environmental factors on the change of microbiome, it would be important to avoid temperature-induced microbiota profile changes for clinical samples. Further investigation using clinical oropharyngeal swabs suggests that the temperature effect on clinical respiratory samples is similar to the effect of intrapersonal sampling variability, though careful estimation is still needed to ensure that the impact caused by temperatures in handling the samples is properly taken into account during data interpretation.

### Availability of supporting data

All sequence data used in the analyses were deposited in Sequence Read Archive (SRA) (http://www.ncbi.nlm.nih.gov/sra) under BioProject PRJNA254831 and SRA accession number SRP044778. Sample IDs, sample information, and basic statistics about the sequences are summarized in Additional file 5: Table S4.

## Abbreviations

16S rDNA: 16S ribosomal RNA gene; cfu: colony-forming unit; DM: Dirichlet multinomial; HMP-R: R statistical software package for HMP; HMPm: Human Microbiome Project; NGS: next-generation sequencing; OTUs: operational taxonomic units; PBS: phosphate-buffered saline; PCoA: principal coordinate analysis; QIIME: Quantitative Insights Into Microbial Ecology; RDP: Ribosomal Database Project; rRNA: ribosomal RNA.

## Competing interests

The authors declare that they have no competing interests.

## Authors' contributions

JH, NZ, RVS, and RAK conceived and designed the experiments. LJM and JMB collected the clinical samples. YY, XXL, and JH performed the experiments. VD, NZ, and JH analyzed the data. RGJ, SJT, RVS, and RAK contributed the reagents/materials/analysis tools. JH, NZ, and VD wrote the manuscript. All authors read, revised, and approved the final manuscript.

## Supplementary Material

Additional file 1: Figure S1 Comparison of extraction methods and swab incubation temperatures. Triplicate Copan flocked swabs spiked with microbial mock community A cells (BEI HM-280) were incubated at 37°C, 4°C, -20°C, or -80°C for 4 weeks, then extracted with MO BIO PowerSoil DNA Isolation Kit or Qiagen DNeasy Blood & Tissue Kit with enzymatic treatment. TaqMan quantitative PCR was used to determine the 16S rDNA copy number in the DNA extracts. Click here for file

Additional file 2: Table S1 Selection of PCR primers for V3 to V1 region of bacterial 16S genes. The primers were compared with all bacterial 16S rRNA genes with length > 1,200 bp in the Ribosomal Database Project (RDP) database. Click here for file

Additional file 3: Table S2 Relative bacterial abundance determined by OTU from 454 pyrosequencing analysis. HM-278D and HM-279D were amplified by PCR for 20 cycles, respectively. HM-280 cells were spiked on FLOQSwabs and stored at -80°C for 4 weeks, then extracted by using Qiagen DNeasy genomic DNA extraction kit or MO BIO PowerSoil genomic DNA extraction kit. HM-280 extracts with Qiagen DNeasy and MO BIO PowerSoil were amplified by PCR for 25 cycles. Data for mock community DNA equal-molar mix used in HMP studies (HMP-MC) were from the HMP Data Analysis and Coordination Center (DACC) and NCBI. All data were analyzed using the QIIME-based pipeline, with classification of operational taxonomic units (OTUs) to bacterial genus level. Click here for file

Additional file 4: Table S3 Genus-based comparison of variations of the mock bacterial community with extraction method and storage temperature. Click here for file

Additional file 5: Table S4 Sample information and sequence reads statistics. Click here for file
