# Supplementary material for: 16S rRNA gene pyrosequencing of reference and clinical samples and investigation of the temperature stability of microbiome profiles
Source: Microbiome. 2014 Sep 16;2:31. doi: 10.1186/2049-2618-2-31 (PMC4165438; doi:10.1186/2049-2618-2-31)
Supplement: Additional file 2: Table S1 — Selection of PCR primers for V3 to V1 region of bacterial 16S genes. The primers were compared with all bacterial 16S rRNA genes with length > 1,200 bp in the Ribosomal Database Project (RDP) database. [file 2049-2618-2-31-S2.pdf]

Table S1 Selection of PCR primers for V3 to V1 region of bacterial 16S genes. The primers were compared with all bacterial 16S rRNA genes with length > 1200 bp in the Ribosomal Database Project (RDP) database.

Primer	Nucleotide Sequence	Exact match		Allow 1 mismatch		Source
		Match/Total	Coverage	Match/Total	Coverage	
**27F1**	AGAGTTTGATCCTGGCTCAG	110916/195967	56.59%	162847/195967	83.10%	HMP protocol
**27F2**	AGAGTTTGATC**M**TGGCTCAG	134274/195967	68.51%	172179/195967	87.86%	This study
**534R**	ATTACCGCGGCTGCTGG	847256/962203	88.05%	954231/962203	99.17%	HMP protocol
**533R**	TTACCGCGGCTGCTGGC	878332/962203	91.28%	955548/962203	99.31%	This study
